# Total shoulder arthroplasty in patients with dementia or mild cognitive impairment

**DOI:** 10.1016/j.jseint.2023.09.004

**Published:** 2023-10-07

**Authors:** Juan Serna, Favian Su, Drew A. Lansdown, Brian T. Feeley, C. Benjamin Ma, Alan L. Zhang

**Affiliations:** Department of Orthopaedic Surgery, University of California San Francisco, San Francisco, CA, USA

**Keywords:** Anatomic total shoulder arthroplasty, Reverse total shoulder arthroplasty, Dementia, Mild cognitive impairment, Surgical complications, Medical complications

## Abstract

**Background:**

Anatomic total shoulder arthroplasty (ATSA) and reverse total shoulder arthroplasty (RTSA) reliably alleviate pain and restore shoulder function for a variety of indications. However, these procedures are not well-studied in patients with neurocognitive impairment. Therefore, the purpose of this study was to investigate whether patients with dementia or mild cognitive impairment (MCI) have increased odds of surgical or medical complications following arthroplasty.

**Methods:**

The PearlDiver database was queried from 2010 through October 2021 to identify a cohort of patients who underwent either ATSA or RTSA and had a minimum 2-year follow-up. Current Procedural Terminology and International Classification of Diseases codes were used to stratify this cohort into three groups: (1) patients with dementia, (2) patients with MCI, and (3) patients with neither condition. Surgical and medical complication rates were compared among these three groups.

**Results:**

The overall prevalence of neurocognitive impairment among patients undergoing total shoulder arthroplasty was 3.0% in a cohort of 92,022 patients. Patients with dementia had increased odds of sustaining a periprosthetic humerus fracture (odds ratio [OR] = 1.46, *P* < .001), developing prosthesis instability (OR = 1.72, *P* < .001), and undergoing revision arthroplasty (OR = 1.55, *P* = .003) after RTSA compared to patients with normal cognition. ATSA patients with dementia did not have an elevated risk of surgical complications or revision. Conversely, RTSA patients with MCI did not have an elevated risk of complications or revision, although ATSA patients with MCI had greater odds of prosthesis instability (OR = 2.51, *P* = .008). Additionally, patients with neurocognitive impairment had elevated odds of medical complications compared to patients with normal cognition, including acute myocardial infarction and cerebrovascular accident.

**Conclusion:**

Compared to patients with normal cognition, RTSA patients with preoperative dementia and ATSA patients with preoperative MCI are at increased risk for surgical complications. Moreover, both ATSA and RTSA patients with either preoperative MCI or dementia are at increased risk for medical complications. As the mean age in the U.S. continues to rise, special attention should be directed towards patients with neurocognitive impairment to minimize postoperative complications aftertotal shoulder arthroplasty, and the risks of this surgery more carefully discussed with patients and their families and caretakers.

The number of total shoulder arthroplasty (TSA) procedures continues to increase worldwide due to an aging population, with the demand for primary arthroplasty projected to increase by at least 300% by 2030.^12^^,^^40^ This trend is most evident in the elderly, for whom utilization has increased at the greatest rate.^40^ Neurocognitive impairment is a relatively common diagnosis in the United States elderly population, with dementia having a prevalence of 10% and mild cognitive impairment (MCI) having a prevalence of 22% in those aged 65 years or older.^32^ Despite this, the outcomes of patients with dementia or MCI undergoing TSA remain poorly characterized.

Most of the orthopedic literature on patients with neurocognitive impairment has been focused on arthroplasty of the lower extremities. Though total hip arthroplasty (THA) and total knee arthroplasty alleviate patient pain and improve quality of life, these procedures result in comparatively worse postoperative functional status and lower independence in activities of daily living (ADLs) in patients with preoperative cognitive impairment.^3^^,^^27^^,^^57^ Lower satisfaction rates among patients with preoperative cognitive impairment may be attributable to these factors, along with a high risk of postoperative delirium that can delay rehabilitation.^53^^,^^55^^,^^57^ Moreover, patients with MCI and dementia are more likely to have gait disorders and increased fall risk, which may contribute to a higher risk of implant failure requiring revision.^2^^,^^19^

Available literature examining shoulder arthroplasty in patients with dementia is scarce and limited to retrospective case series or short-term perioperative outcomes.^4^ Furthermore, the outcomes of patients with MCI after TSA have not yet been studied. As the prevalence of dementia is projected to double by 2050, it will be increasingly important to characterize the risks associated with shoulder arthroplasty among patients with neurocognitive impairment.^38^ Therefore, the purpose of this study is to use a national claims database to evaluate the surgical and medical complications of patients with neurocognitive impairment undergoing TSA. We hypothesized that patients with cognitive impairment would have increased complication rates compared to patients with normal cognition, with increasing rates observed in patients with more severe impairment.

## Methods

### Study design

A retrospective cohort study was performed using the PearlDiver software (PearlDiver, Colorado Springs, CO, USA) to query the Mariner database.^46^ Mariner is an administrative claims database of private and public payers in the United States that comprises over 157 million individuals and longitudinally tracks individual patient diagnoses and procedures.^46^ The database was queried from 2010 to October 2021 to identify patient cohorts. This study utilizes deidentified data and was exempt from Institutional Review Board approval at our institution.

### Patient selection

**Figure 1:**
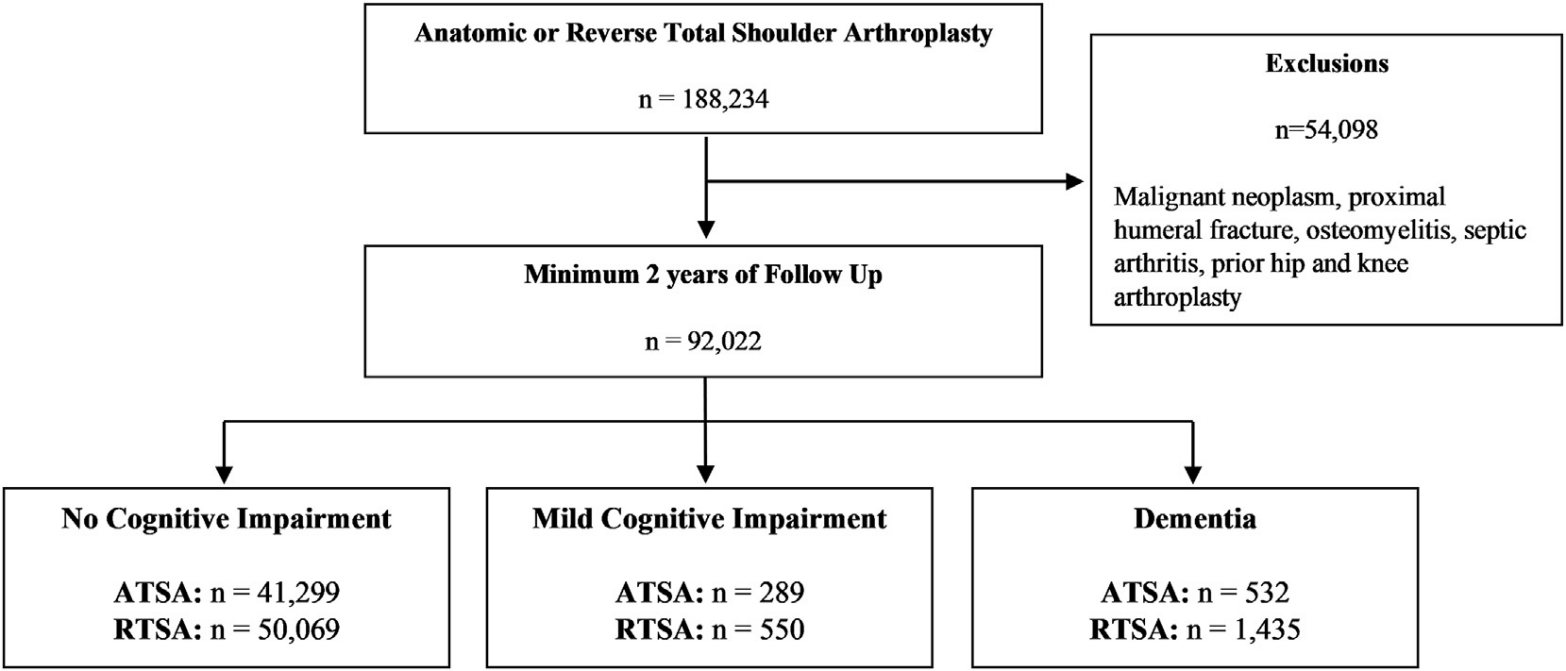
Strengthening of the reporting of observational studies in epidemiology (STROBE) diagram depicting selection and grouping of patients included in this study. *ATSA*, anatomic total shoulder arthroplasty; *RTSA,* reverse total shoulder arthroplasty.

International Classification of Diseases (ICD), Ninth and Tenth Revision codes were used to identify patients who underwent either a primary anatomic total shoulder arthroplasty [ATSA] (9th: 81.80; 10th: 0RRJ0JZ, 0RRK0JZ) or reverse total shoulder arthroplasty [RTSA] (9th: 81.88; 10th: 0RRJ00Z, 0RRK00Z) with minimum follow-up of 2-years (Fig. 1). Patients with history of septic arthritis or osteomyelitis of the shoulder, malignant neoplasm of the upper extremity, or proximal humeral fracture were excluded. Patients with THA and total knee arthroplasty before or after TSA were also excluded due to lack of ICD-9 and 10 complication codes specific to the shoulder.

To determine the effects of the severity of preoperative cognitive impairment, patients were divided into three groups (1: normal cognition, 2: MCI, and 3: dementia) using ICD-9 and 10 codes. Patients who had records of both MCI and dementia were categorized as having dementia given that neurocognitive impairment is a progressive disease.

### Identification of patient comorbidities

Comorbid conditions were identified using ICD-9 and ICD-10 codes (Supplementary Table S1). Any patient whose medical history included one of the diagnostic codes for a comorbidity within a year prior to surgery was classified as having that respective comorbidity. Factors that have been reported in the literature to affect cognition in this patient population—including anxiety, depression, and psychiatric disorders—were also assessed.^23^^,^^30^^,^^31^^,^^37^ Chronic use of oral sleep aids (zolpidem tartrate, eszopiclone) was defined as having multiple prescriptions filled in the past year and at least one in the 3 months leading up to surgery.

### Identification of surgical complications, medical complications, and revision arthroplasty

Surgical complications were tracked for a minimum of two years and included shoulder prosthetic joint infection, prosthesis instability, component loosening, periprosthetic fracture, and revision shoulder arthroplasty (Supplementary Table S2). Medical complications were tracked for 90 days after surgery using ICD-9 and 10 codes (Supplementary Table S3). Postoperative delirium and discharge to a skilled nursing facility (SNF) were tracked for 7 days after surgery.

### Statistical analysis

**Figure 2:**
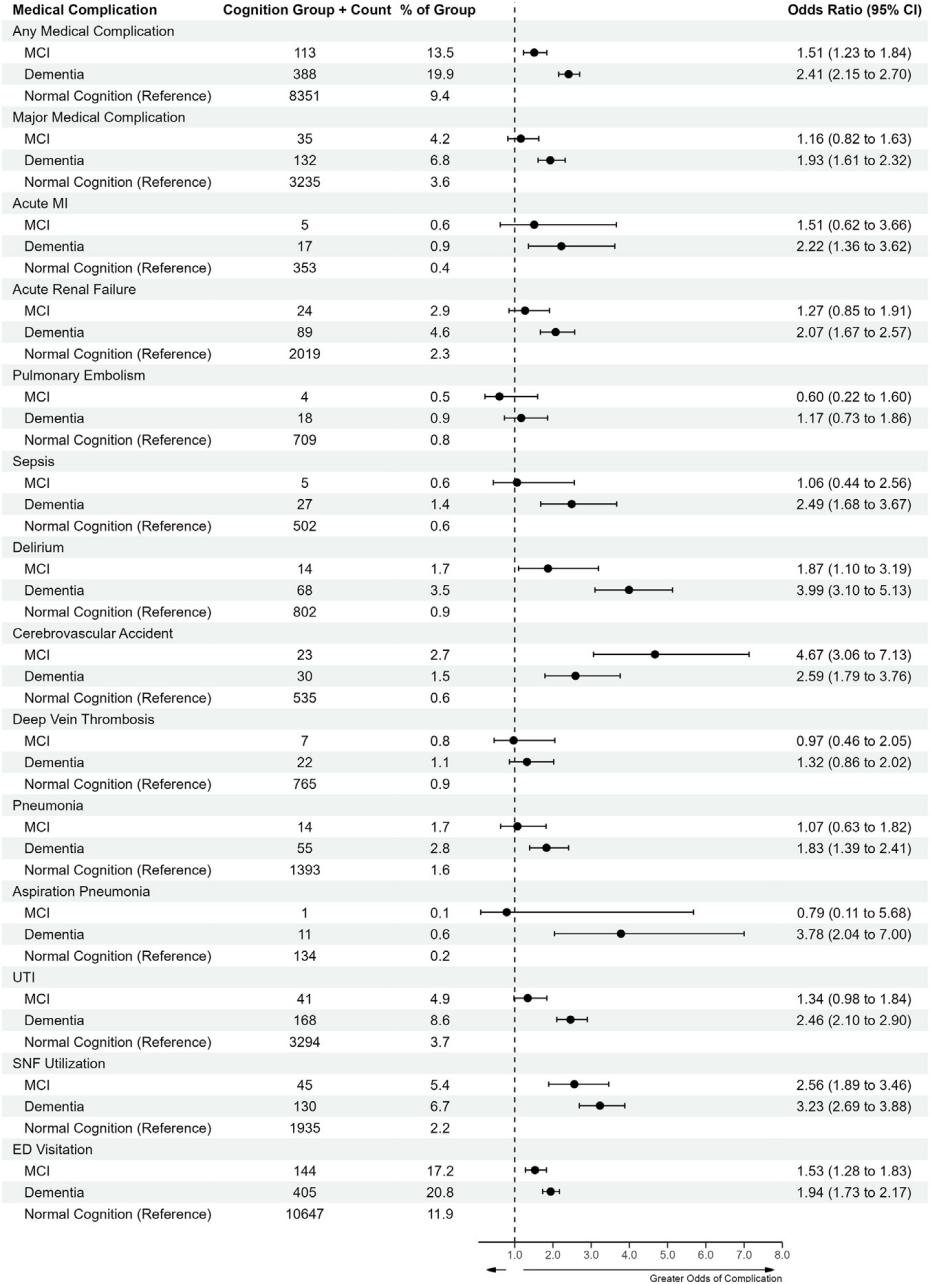
Forest Plot of Medical Complications in Total Shoulder Arthroplasty Patients. *CI*, confidence interval; *MCI*, mild cognitive impairment; *MI*, myocardial infarction; *UTI*, urinary tract infection; *SNF*, skilled nursing facility; *ED*, emergency department. Data is in aggregate for both anatomic and reverse total shoulder arthroplasty. Major medical complication defined as: acute MI, acute renal failure, pulmonary embolism, sepsis. SNF utilization and ED visitation are not considered in the overall medical complication rate.

All statistical analyses were performed using R statistical software version 4.1 integrated with PearlDiver (R Foundation for Statistical Computing, Vienna, Austria). Descriptive statistics including means, standard deviations, frequency, and proportions were reported as appropriate. Bivariate analyses were performed using Fisher’s exact or Chi-square testing for categorical variables and One-way analysis of variance testing for continuous variables. Multivariate logistic regression models were performed to assess the impact of covariates on postoperation surgical complications following ATSA and RTSA. Covariates with clinical relevance based on previous literature were entered into the model and removed with a backward selection method. Covariates included ADL dependence, psychotic disorders, other psychiatric conditions, depression, anxiety, osteoporosis, age, gender, tobacco use disorder and chronic oral sleep aid use. Results were reported as adjusted odds ratios (ORs) with 95% confidence intervals (CIs). Significance was set at *P* < .05. The forest plot in Fig. 2 was made using the R package “Forester.”.^5^

## Results

### Patient demographics

Demographics of patients undergoing TSA are shown in Table I. There were 42,120 patients who underwent ATSA and 52,054 patients who underwent RTSA (Fig. 1). Of these, 2.0% and 3.8% of patients had some degree of neurocognitive impairment in the ATSA and RTSA groups, respectively. Patients with dementia (72.7 ± 6.2 years) were significantly older than both patients with MCI (70.4 ± 7.5 years, *P* < .001) and normal cognition (67.8 ± 8.3 years, *P* < .001). Patients with dementia also had higher Charlson Comorbidity Index scores (3.7 ± 3.2) than both patients with MCI (3.3 ± 3.0, *P* < .001) and normal cognition (2.0 ± 2.4, *P* < .001).

**Table I:** Patient demographics.

	Dementia (n = 1947)	MCI (n = 839)	Normal cognition (n = 89,236)
Age	72.7 (6.2)	70.4 (7.5)	67.8 (8.3)
CCI	3.7 (3.2)	3.3 (3.0)	2.0 (2.4)
Female gender	1253 (64.4%)	480 (57.2%)	49,300 (55.2%)
Obesity	286 (14.7%)	162 (19.3%)	16,208 (18.2%)
Chronic oral sleep medication use	91 (4.67%)	44 (5.2%)	4944 (5.5%)
Comorbid conditions			
Anxiety	546 (28.0%)	244 (29.1%)	15,353 (17.2%)
Depression	779 (40.0%)	327 (39.0%)	19,575 (21.9%)
Psychotic disorders	91 (4.7%)	13 (1.6%)	674 (0.8%)
Other psychiatric conditions	118 (6.1%)	26 (3.1%)	1833 (2.1%)
Osteoporosis	284 (14.6%)	99 (11.8%)	7949 (8.9%)
Tobacco use disorder	487 (25.0%)	269 (32.1%)	20,821 (23.3%)

*MCI*, mild cognitive impairment; *CCI*, Charlson comorbidity index.

Data expressed as count (%) or mean (standard deviation). Psychotic disorders includes: schizophrenic disorders, schizoaffective disorders, delusional disorders. Other psychiatric conditions include episodic mood disorders.

Patients with dementia had higher preoperative rates of anxiety, depression, psychotic disorders and other psychiatric conditions, and osteoporosis compared to other groups.

### Surgical complications

Postoperative surgical complications for patients undergoing ATSA and RTSA are shown in Tables II and III, respectively. Among both patients undergoing ATSA and RTSA, the three cognition subgroups differed significantly in postoperative rates of prosthesis instability and periprosthetic fracture.

**Table II:** ATSA surgical complications.

	Dementia (n = 532)	MCI (n = 289)	Normal cognition (n = 41,299)	*P* value
Prosthetic joint infection	8 (1.5%)	6 (2.1%)	547 (1.3%)	.410
Prosthesis instability	11 (2.1%)	9 (3.1%)	518 (1.3%)	**.008**
Component loosening	7 (1.3%)	5 (1.7%)	444 (1.1%)	.371
Periprosthetic fracture	11 (2.1%)	3 (1.0%)	393 (1.0%)	**.039**
Revision arthroplasty	15 (2.8%)	8 (2.8%)	804 (2.0%)	.172

*ATSA*, anatomic total shoulder arthroplasty; *MCI*, mild cognitive impairment.

Data expressed as count (%). Bolded *P* values denote significance.

**Table III:** RTSA surgical complications.

	Dementia (n = 1435)	MCI (n = 550)	Normal cognition (n = 50,069)	*P* value
Prosthetic joint infection	22 (1.5%)	11 (2.0%)	922 (1.8%)	.663
Prosthesis instability	65 (4.5%)	21 (3.8%)	1378 (2.8%)	**<.001**
Component loosening	27 (1.9%)	4 (0.7%)	758 (1.5%)	.167
Periprosthetic fracture	91 (6.3%)	26 (4.7%)	1921 (3.8%)	**<.001**
Revision arthroplasty	53 (3.7%)	16 (2.9%)	1369 (2.7%)	.090

*RTSA*, reverse total shoulder arthroplasty; *MCI*, mild cognitive impairment.

Data expressed as count (%). Bolded *P* values denote significance.

More specifically, in patients undergoing ATSA, dementia was associated with a significantly increased odds of experiencing a periprosthetic fracture compared to patients with normal cognition [OR = 2.20 (95% CI: 1.20-4.03), *P* = .039]. Similarly, patients with MCI who underwent ATSA were significantly more likely to sustain a dislocation [OR = 2.53 (95% CI: 1.30-4.94), *P* = .012]. In patients undergoing RTSA, dementia was likewise associated with increased odds of periprosthetic fracture compared to patients with normal cognition [OR = 1.70 (95% CI: 1.37-2.11), *P* < .001]; in addition, these patients were also more likely to sustain a dislocation [OR = 1.68 (95% CI: 1.30-2.16), *P* < .001] and require revision arthroplasty [OR = 1.36 (95% CI: 1.03-1.80), *P* = .029]. However, there was no difference in surgical complication rates between patients with MCI and normal cognition undergoing RTSA.

### Medical complications

The rates of postoperative medical complications for TSA patients are shown in Fig. 2. Overall, patients with dementia [OR = 2.41 (95% CI: 2.15-2.70), *P* < .001] or MCI [OR = 1.51 (95% CI: 1.23-1.84), *P* < .001] who underwent TSA had greater odds of experiencing any medical complication compared to patients with normal cognition. During the postoperative hospitalization, patients with dementia were more likely to experience delirium and be discharged to SNF compared to patients with normal cognition (*P* < .001). At 90-days, patients with dementia were more likely to have a visitation to the emergency department (ED) or experience an acute myocardial infarction (MI), cerebrovascular accident (CVA), sepsis, pneumonia, aspiration pneumonia, acute renal failure or urinary tract infection (*P* < .001). Patients with MCI likewise were significantly more likely to experience postoperative delirium (*P* = .019), be discharged to an SNF, have an ED visit, and experience a CVA (*P* < .001).

### Multivariate analysis of postsurgical complications in TSA patients

On multivariate analysis, there was no difference in ATSA complication rates between patients with dementia and normal cognition (Table IV). Interestingly, patients with MCI had greater odds of prosthesis instability compared to patients with normal cognition [OR = 2.51 (95% CI: 1.18-4.63, *P* = .008)].

**Table IV:** Multivariate analysis of surgical complications in patients with dementia, mild cognitive impairment, and normal cognition undergoing anatomic or reverse total shoulder arthroplasty.

Predictor	Prosthesis instability		Periprosthetic fracture		Revision arthroplasty	
	OR (95% CI)	*P* value	OR (95% CI)	*P* value	OR (95% CI)	*P* value
ATSA patients						
Dementia	1.70 (0.87, 2.99)	.091	1.65 (0.84, 2.92)	.112	1.43 (0.81, 2.33)	.186
Mild cognitive impairment	2.51 (1.18, 4.63)	**.008**	0.93 (0.23, 2.45)	.898	1.39 (0.63, 2.64)	.366
Normal cognition	Reference	--	Reference	--	Reference	--
RTSA patients						
Dementia	1.72 (1.31, 2.22)	**<.001**	1.46 (1.16, 1.82)	**<.001**	1.55 (1.15, 2.04)	**.003**
Mild cognitive Impairment	1.43 (0.89, 2.17)	.111	1.25 (0.82, 1.82)	.279	1.11 (0.64, 1.78)	.681
Normal cognition	Reference	--	Reference	--	Reference	--

*OR*, odds ratio; *CI*, confidence interval; *ATSA*, anatomic total shoulder arthroplasty; *RTSA*, reverse total shoulder arthroplasty.

Bolded *P* values denote significance.

Similarly, RTSA patients with dementia were more likely to sustain a dislocation [OR = 1.72 (95% CI: 1.31-2.22), *P* < .001], develop a periprosthetic fracture [OR = 1.46 (95% CI: 1.16-1.82), *P* < .001] or require revision arthroplasty [OR = 1.55 (95% CI: 1.15-2.04), *P* = .003] compared to patients with normal cognition. However, RTSA patients with MCI did not have an increased risk of complications compared to patients with normal cognition.

## Discussion

In the present study, the overall prevalence of TSA patients with neurocognitive impairment was 3.0%. These patients had a higher rate of surgical complications, medical complications, and health-care utilization, including postoperative discharge to SNF and 90-day ED visits. Moreover, patients with more advanced disease had the greatest risk of surgical complications after RTSA. As the mean age in the U.S. increases, it is likely that not only will the frequency of TSA in general rise, but so too will the number of patients with dementia and MCI undergoing the procedure.^59^ Thus, it will be increasingly important for both patients and their families and providers to be aware of the surgical and medical complications associated with TSA.

Preexisting dementia in patients was associated with increased odds of postoperative periprosthetic humerus fracture (PPF). Patients with dementia undergoing ATSA experienced a periprosthetic fracture at an incidence of 2.1% compared to 1.0% in patients with normal cognition. Likewise, patients with dementia undergoing RTSA sustained a periprosthetic fracture at an incidence of 6.3% compared to 3.8% in patients with normal cognition. Similar findings have been observed in patients with other neurocognitive disorders such as Parkinson’s Disease (PD). In a case-control study of 4199 TSA patients with PD, Burrus et al showed an increased risk of humerus fractures compared to controls at 1-year.^7^ Several studies in the hip and knee literature have shown similar trends, including a higher rate of periprosthetic hip fracture in patients with dementia undergoing THA.^1^^,^^19^^,^^18^ The higher rates of postoperative fracture may be due to the increased gait instability and higher fall risk among patients with neurocognitive impairment.^2^^,^^17^ Furthermore, these patients have higher rates of osteoporosis due to decreased physical activity and greater dependence on others for ADLs.^11^^,^^15^^,^^52^^,^^60^^,^^61^ The higher rates of PPF in patients with neurocognitive impairment may consequently contribute to a higher risk of revision.^7^^,^^19^ Periprosthetic humeral fractures are devastating to the patient as many of them use assistive devices and require their upper extremity to ambulate. Preoperative visual acuity and home evaluations as well as referrals to a neurologist to maximize disease control should be offered to patients to minimize the risk of falls.^9^

The higher rates of prosthesis instability may be attributed to the lack of neuromuscular control among patients with neurocognitive disease.^7^^,^^9^^,^^24^^,^^34^ Moreover, these patients may be less compliant with postoperative immobilization, as a shoulder immobilizer may be perceived as a restraint. A systematic review of patients with PD showed higher rates of prosthesis instability compared to normal cognition, with one included study placing incidence at 4.0% and 5.6% for these patients when undergoing ATSA or RTSA, respectively.^7^^,^^9^ A recent retrospective cohort study by Papalia et al also demonstrated higher risk of shoulder dislocation in patients with PD compared to controls.^42^ As with PPF, risk of prosthesis instability can contribute to the observed elevated rates of revision arthroplasty. Given the higher rate of instability in patients with cognitive disorders, special consideration should be directed towards mitigating this risk at the time of the index procedure. One possible option is to use a constrained liner, which potentially improves stability without sacrificing motion.^16^ However, a cause for concern with these constrained liners is the greater wear and volume loss they undergo compared to nonconstrained liners, which can potentially lead to aseptic loosening as a long-term complication.^8^^,^^16^ RTSA with subscapularis repair should also be considered, as prior studies have shown reduced dislocation rates.^35^ We do not routinely recommend lateralizing or distalizing the glenoid due to the risk of acromial stress fracture in this older population with a high prevalence of osteoporosis.^25^ However, the surgeon may consider humeral lateralization to increase the deltoid wrapping effect and potentially increase stability.^28^^,^^44^^,^^51^

Postsurgical complications in patients with MCI, a population that to our knowledge has not been studied for longitudinal outcomes following TSA, were less pronounced than for dementia. Patients with MCI undergoing RTSA did not have a statistically significantly increased risk of prosthesis instability, periprosthetic fracture or revision compared to patients with normal cognition. This may suggest that there is a cognitive threshold at which the risk of complication increases, with effects potentially becoming more severe with greater cognitive impairment. Unfortunately, due to the lack of specificity of ICD codes, we were not able to categorize the severity of dementia. However, ATSA patients with MCI did have higher odds of prosthesis instability compared to patients with normal cognition, whereas patients with dementia did not. This may be due to insufficient power, as there was a trend towards significance among patients with dementia. The higher rate of instability after ATSA may be attributed to decreased compliance with postoperative immobilization, resulting in subscapularis failure.^14^^,^^26^

Unsurprisingly, patients with neurocognitive impairment also have a higher rate of medical complications following TSA, including acute MI, sepsis and CVA. The overall rate of medical complications was 19.9%, 13.5% and 9.4% in patients with dementia, MCI and normal cognition, respectively. Patients with dementia moreover had 93% greater odds of experiencing a major medical complication (acute MI, acute renal failure, pulmonary embolism, or sepsis) compared to patients with normal cognition. The increased rates of medical complications may have to do with reduced ability to communicate symptoms by patients with neurocognitive impairment.^22^^,^^29^^,^^49^ Additionally, risk factors including dysphagia and anti-psychotic use in patients with dementia may also contribute to medical complications following TSA.^21^^,^^22^^,^^29^^,^^41^^,^^43^^,^^48^^,^^54^ Importantly, we found significantly higher rates of postoperative delirium in patients with either dementia or MCI compared to patients with normal cognition. These findings are consistent with prior hip and knee literature in patients with cognitive impairment.^19^^,^^18^^,^^29^^,^^62^ The high rates of observed postoperative delirium are problematic for recovery following TSA, and especially so for patients who undergo ATSA since increased noncompliance with a brace can stress repair of the subscapularis.^14^^,^^26^ Delirium rates should, therefore, be mitigated through methods focused on prevention and early management, including early mobilization and appropriate analgesia such as regional nerve blocks.^20^^,^^26^^,^^56^

Although TSA can reliably restore function and alleviate pain in patients with cognitive impairment, these patients are at an increased risk of postoperative surgical complications—such as revision, prosthesis instability and periprosthetic fracture—and medical complications, such as sepsis. The risk of revision shoulder arthroplasty in patients with dementia undergoing ATSA or RTSA was 2.8% and 3.7%, respectively, in this cohort. These rates are similar to those seen in patients with BMI over 30 kg/m^2^ and hemoglobin A1c greater than 7.0%, though slightly lower than in patients with opioid dependence.^6^^,^^10^^,^^36^ Similarly, the risk of periprosthetic fracture in patients with dementia undergoing ATSA or RTSA was 2.1% and 6.3%, respectively, in this study, which is higher than seen in patients with BMI over 30 kg/m^2^, metabolic syndrome (including A1c >6.5%), or opioid dependence.^6^^,^^10^^,^^33^ Finally, we found that patients with dementia had a dislocation risk of 2.1% in ATSA and 4.5% in RTSA. These findings for ATSA are similar to those seen in patients with BMI >30 kg/m^2^ and A1c >6.5%, but higher than in patients with opioid dependence; for RTSA patients, the risk of instability is higher compared to all three of these other comorbid conditions.^6^^,^^10^^,^^13^

To minimize postoperative complications in patients with cognitive impairment, increased focus should be placed on mitigating factors, including delirium management and measures to minimize fall risk. Care teams should attempt to achieve minimal depth of intraoperative sedation while also ensuring adequate preoperative and postoperative anesthesia, as such measures can mitigate postoperative delirium.^50^ Similarly, physicians should avoid overprescribing narcotics to prevent oversedation; multimodal management should instead be utilized to control postoperative pain.^45^ Thus, in the perioperative period, a team-based approach involving the orthopedic surgeons, geriatric specialists, physiotherapists and pain management specialists should be considered to manage the acute postoperative care of these patients^19^^,^^29^^,^^50^ Regarding fall risk, comprehensive preoperative assessment of patient cognition as well as home evaluation of risks can help to mitigate falls.^29^^,^^47^ Moreover, patient sensory and mobility interventions, including corrective lenses, hearing aids, mobility aids and improved footwear, are also beneficial to prevent falls.^39^^,^^58^

The present study has several limitations. The major limitation is that it was a retrospective study utilizing the large PearlDiver administrative database, meaning that accurate results are dependent on the quality and accuracy of billing codes. Miscoding or noncoding by providers may introduce error and decrease the power of the study. Additionally, some codes may be nonspecific to shoulder arthroplasty only, though we attempted to maximize specificity for TSA by excluding other total joint arthroplasties preceding and following TSA. Moreover, information about prosthesis design for RTSA could not be determined, and the effect of factors that increase stability, such as lateralization or polyethylene thickness, could not be adjusted for in our analysis. Another limitation was the small number of patients with neurocognitive impairment who underwent ATSA and RTSA. As such, we may have been insufficiently powered to detect a difference in complications between groups. This may explain why multivariate analysis found elevated rates of complications only for patients with MCI and not dementia in the ATSA cohort, as there was a trend toward significance.

## Conclusion

Compared to patients with normal cognition, RTSA patients with preoperative dementia and ATSA patients with preoperative MCI are at increased risk for surgical complications. Moreover, both ATSA and RTSA patients with either preoperative MCI or dementia are at increased risk for medical complications. As the mean age in the U.S. increases and the prevalence of both MCI and dementia rises, special attention will need to be directed towards patients with neurocognitive impairment undergoing total shoulder arthroplasty.

## Disclaimers

Funding: No funding was disclosed by the authors.

Conflict of interest: Favian Su: This author receives funding from the National Institute on Aging. Drew A. Lansdown: This author is a consultant for Vericel Inc and AlloSource. He is an editorial board member of Arthroscopy and Journal of Cartilage and Joint Preservation. Brian T. Feeley: This author receives grant support from Orthofix inc, NIH, CIRM, VAHealthcare System; stocks from Bionik and Kaliber.ai. He is an editorial board member of Journal of Shoulder and Elbow Surgery and the Current Reviews in Musculoskeletal Medicine. C. Benjamin Ma: This author receives grant support from Aesculap Inc, Zimmer Biomet, and the NIH. He is a consultant for Stryker and CONMED Linvatec. He also receives royalties from CONMED Linvatec. Alan L. Zhang: This author is a consultant for DePuy Mitek Inc and Stryker Corporation. He is an editorial board member of American Journal of Sports Medicine and Arthroscopy. However, all authors, their immediate families, and any research foundation with which they are affiliated did not receive any financial payments or other benefits from any commercial entity related to the subject of this article.
