# Supplementary material for: Total shoulder arthroplasty in patients with dementia or mild cognitive impairment
Source: JSES Int. 2023 Oct 7;8(1):159–66. doi: 10.1016/j.jseint.2023.09.004 (PMC10837705; doi:10.1016/j.jseint.2023.09.004)
Supplement: Supplementary Table S1 [file mmc1.docx]

| **Supplementary Table I: ICD-9 and ICD-10 Codes Queried for Comorbidities** | |
| --- | --- |
| **Description** | **Codes Queried** |
| Obesity | ICD-9-D-V8530, ICD-9-D-V8531, ICD-9-D-V8532, ICD-9-D-V8533, ICD-9-D-V8534, ICD-9-D-V8535, ICD-9-D-V8536, ICD-9-D-V8537, ICD-9-D-V8538, ICD-9-D-V8539, ICD-9-D-V8541, ICD-9-D-V8542, ICD-9-D-V8543, ICD-9-D-V8544, ICD-9-D-V8545  ICD-10-D-Z6830, ICD-10-D-Z6831, ICD-10-D-Z6832, ICD-10-D-Z6833, ICD-10-D-Z6834, ICD-10-D-Z6835, ICD-10-D-Z6836, ICD-10-D-Z6837, ICD-10-D-Z6838, ICD-10-D-Z6839, ICD-10-D-Z6841, ICD-10-D-Z6842, ICD-10-D-Z6843, ICD-10-D-Z6844, ICD-10-D-Z6845 |
| Chronic Oral Sleep Medication Use | GENERIC_DRUG-ZOLPIDEM_TARTRATE, GENERIC_DRUG-ESZOPICLONE, DRUG-AMBIEN, DRUG-AMBIEN_CR, DRUG-LUNESTA |
| Anxiety | ICD-9-D-30000, ICD-9-D-30001, ICD-9-D-30002, ICD-9-D-30009  ICD-10-D-F41.0, ICD-10-D-F41.1, ICD-10-D-F41.3, ICD-10-D-F41.8, ICD-10-D-F41.9 |
| Depression | ICD-9-D-29620, ICD-9-D-29621, ICD-9-D-29622, ICD-9-D-29623, ICD-9-D-29624, ICD-9-D-29625, ICD-9-D-29626, ICD-9-D-29630, ICD-9-D-29631, ICD-9-D-29632, ICD-9-D-29633, ICD-9-D-29634, ICD-9-D-29635, ICD-9-D-29636, ICD-9-D-29682, ICD-9-D-3004, ICD-9-D-3090, ICD-9-D-3091, ICD-9-D-30112, ICD-9-D-311, ICD-9-D-29620, ICD-9-D-29621, ICD-9-D-29622, ICD-9-D-29623, ICD-9-D-29624, ICD-9-D-29625, ICD-9-D-29626, ICD-9-D-29630, ICD-9-D-29631, ICD-9-D-29632, ICD-9-D-29633, ICD-9-D-29634, ICD-9-D-29635, ICD-9-D-29636, ICD-9-D-29682  ICD-10-D-F320, ICD-10-D-F321, ICD-10-D-F322, ICD-10-D-F323, ICD-10-D-F324, ICD-10-D-F325, ICD-10-D-F3281, ICD-10-D-F3289, ICD-10-D-F329, ICD-10-D-F32A, ICD-10-D-F330, ICD-10-D-F331, ICD-10-D-F332, ICD-10-D-F333, ICD-10-D-F3340, ICD-10-D-F3341, ICD-10-D-F3342, ICD-10-D-F338, ICD-10-D-F339 |
| Psychotic Disorders | ICD-9-D-29500, ICD-9-D-29501, ICD-9-D-29502, ICD-9-D-29503, ICD-9-D-29504, ICD-9-D-29505, ICD-9-D-29510, ICD-9-D-29511, ICD-9-D-29512, ICD-9-D-29513, ICD-9-D-29514, ICD-9-D-29515, ICD-9-D-29520, ICD-9-D-29521, ICD-9-D-29522, ICD-9-D-29523, ICD-9-D-29524, ICD-9-D-29525, ICD-9-D-29530, ICD-9-D-29531, ICD-9-D-29532, ICD-9-D-29533, ICD-9-D-29534, ICD-9-D-29535, ICD-9-D-29540, ICD-9-D-29541, ICD-9-D-29542, ICD-9-D-29543, ICD-9-D-29544, ICD-9-D-29545, ICD-9-D-29550, ICD-9-D-29551, ICD-9-D-29552, ICD-9-D-29553, ICD-9-D-29554, ICD-9-D-29555, ICD-9-D-29560, ICD-9-D-29561, ICD-9-D-29562, ICD-9-D-29563, ICD-9-D-29564, ICD-9-D-29565, ICD-9-D-29570, ICD-9-D-29571, ICD-9-D-29572, ICD-9-D-29573, ICD-9-D-29574, ICD-9-D-29575, ICD-9-D-29580, ICD-9-D-29581, ICD-9-D-29582, ICD-9-D-29583, ICD-9-D-29584, ICD-9-D-29585, ICD-9-D-29590, ICD-9-D-29591, ICD-9-D-29592, ICD-9-D-29593, ICD-9-D-29594, ICD-9-D-29595, ICD-9-D-2970, ICD-9-D-2971, ICD-9-D-2972, ICD-9-D-2973, ICD-9-D-2983, ICD-9-D-2984, ICD-9-D-2988, ICD-9-D-2989  ICD-10-D-F200, ICD-10-D-F201, ICD-10-D-F202, ICD-10-D-F203, ICD-10-D-F205, ICD-10-D-F2081, ICD-10-D-F2089, ICD-10-D-F209, ICD-10-D-F250, ICD-10-D-F251, ICD-10-D-F258, ICD-10-D-F259, ICD-10-D-F28, ICD-10-D-F29, ICD-10-D-F22, ICD-10-D-F23, ICD-10-D-F24 |
| Other Psychiatric Conditions | ICD-9-D-29600, ICD-9-D-29601, ICD-9-D-29602, ICD-9-D-29603, ICD-9-D-29604, ICD-9-D-29605, ICD-9-D-29606, ICD-9-D-29610, ICD-9-D-29611, ICD-9-D-29612, ICD-9-D-29613, ICD-9-D-29614, ICD-9-D-29615, ICD-9-D-29616, ICD-9-D-29640, ICD-9-D-29641, ICD-9-D-29642, ICD-9-D-29643, ICD-9-D-29644, ICD-9-D-29645, ICD-9-D-29646, ICD-9-D-29650, ICD-9-D-29651, ICD-9-D-29652, ICD-9-D-29653, ICD-9-D-29654, ICD-9-D-29655, ICD-9-D-29656, ICD-9-D-29660, ICD-9-D-29661, ICD-9-D-29662, ICD-9-D-29663, ICD-9-D-29664, ICD-9-D-29665, ICD-9-D-29666, ICD-9-D-2967, ICD-9-D-29680, ICD-9-D-29681, ICD-9-D-29689  ICD-10-D-F310, ICD-10-D-F3110, ICD-10-D-F3111, ICD-10-D-F3112, ICD-10-D-F3113, ICD-10-D-F312, ICD-10-D-F3130, ICD-10-D-F3131, ICD-10-D-F3132, ICD-10-D-F314, ICD-10-D-F315, ICD-10-D-F3160, ICD-10-D-F3161, ICD-10-D-F3162, ICD-10-D-F3163, ICD-10-D-F3164, ICD-10-D-F3170, ICD-10-D-F3171, ICD-10-D-F3172, ICD-10-D-F3173, ICD-10-D-F3174, ICD-10-D-F3175, ICD-10-D-F3176, ICD-10-D-F3177, ICD-10-D-F3178, ICD-10-D-F3181, ICD-10-D-F3189, ICD-10-D-F319 |
| Osteoporosis | ICD-9-D-73300, ICD-9-D-73301, ICD-9-D-73302, ICD-9-D-73303, ICD-9-D-73309, ICD-9-D-7337  ICD-10-D-M810, ICD-10-D-M816, ICD-10-D-M818, ICD-10-D-Z743, ICD-10-D-Z749 |
| Tobacco Use Disorder | ICD-9-D-3051  ICD-10-D-F17200, ICD-10-D-F17203, ICD-10-D-F17208, ICD-10-D-F17209, ICD-10-D-F17210, ICD-10-D-F17213, ICD-10-D-F17218, ICD-10-D-F17219, ICD-10-D-F17220, ICD-10-D-F17223, ICD-10-D-F17228, ICD-10-D-F17229, ICD-10-D-F17290, ICD-10-D-F17293, ICD-10-D-F17298, ICD-10-D-F17299, ICD-10-D-Z720, ICD-10-D-Z87891 |
| ADL Dependence | ICD-9-D-E0140, ICD-9-D-E0141, ICD-9-D-E0149  ICD-10-D-Y93F1, ICD-10-D-Y93F2, ICD-10-D-Y93F9, ICD-10-D-Z736, ICD-10-D-Z741, ICD-10-D-Z742, ICD-10-D-Z743, ICD-10-D-Z749 |
| Codes reported with decimal points removed, per PearlDiver coding format. ADL, Activities of Daily Living. | |
