# Supplementary material for: Total shoulder arthroplasty in patients with dementia or mild cognitive impairment
Source: JSES Int. 2023 Oct 7;8(1):159–66. doi: 10.1016/j.jseint.2023.09.004 (PMC10837705; doi:10.1016/j.jseint.2023.09.004)
Supplement: Supplementary Table S2 [file mmc2.docx]

| **Supplementary Table II: ICD-9, ICD-10 and CPT Codes Queried for Surgical Complications** | |
| --- | --- |
| **Description** | **Codes Queried** |
| Prosthetic Joint Infection | ICD-9-D-99666, ICD-9-D-99667, ICD-9-D-99851, ICD-9-D-71101, ICD-9-D-71191  ICD-10-D-T8450XA, ICD-10-D-T8459XA, ICD-10-D-T847XXA, ICD-10-D-M00011, ICD-10-D-M00012, ICD-10-D-M00019, ICD-10-D-M00111, ICD-10-D-M00112, ICD-10-D-M00119, ICD-10-D-M00211, ICD-10-D-M00212, ICD-10-D-M00219, ICD-10-D-M00811, ICD-10-D-M00812, ICD-10-D-M00819 |
| Prosthesis Instability | ICD-9-D-71831, ICD-9-D-83100, ICD-9-D-83101, ICD-9-D-83102, ICD-9-D-83103, ICD-9-D-83109  ICD-10-D-M24411, ICD-10-D-M24412, ICD-10-D-M24419, ICD-10-D-S43001A, ICD-10-D-S43002A, ICD-10-D-S43003A, ICD-10-D-S43004A, ICD-10-D-S43005A, ICD-10-D-S43006A, ICD-10-D-S43011A, ICD-10-D-S43012A, ICD-10-D-S43013A, ICD-10-D-S43014A, ICD-10-D-S43015A, ICD-10-D-S43016A, ICD-10-D-S43021A, ICD-10-D-S43022A, ICD-10-D-S43023A, ICD-10-D-S43024A, ICD-10-D-S43025A, ICD-10-D-S43026A, ICD-10-D-S43031A, ICD-10-D-S43032A, ICD-10-D-S43033A, ICD-10-D-S43034A, ICD-10-D-S43035A, ICD-10-D-S43036A, ICD-10-D-S43081A, ICD-10-D-S43082A, ICD-10-D-S43083A, ICD-10-D-S43084A, ICD-10-D-S43085A, ICD-10-D-S43086A |
| Component Loosening | ICD-9-D-99641, ICD-9-D-99643  ICD-10-D-M89512, ICD-10-D-M89511, ICD-10-D-T84038A, ICD-10-D-T84039A |
| Periprosthetic Fracture | ICD-9-D-81200, ICD-9-D-81201, ICD-9-D-81209, ICD-9-D-81221  ICD-10-D-M9731XA, ICD-10-D-M9732XA, ICD-10-D-S42201A, ICD-10-D-S42202A, ICD-10-D-S42209A, ICD-10-D-S42211A, ICD-10-D-S42212A, ICD-10-D-S42213A, ICD-10-D-S42214A, ICD-10-D-S42215A, ICD-10-D-S42216A, ICD-10-D-S42221A, ICD-10-D-S42222A, ICD-10-D-S42223A, ICD-10-D-S42224A, ICD-10-D-S42225A, ICD-10-D-S42226A, ICD-10-D-S42231A, ICD-10-D-S42232A, ICD-10-D-S42239A, ICD-10-D-S42241A, ICD-10-D-S42242A, ICD-10-D-S42249A, ICD-10-D-S42291A, ICD-10-D-S42292A, ICD-10-D-S42293A, ICD-10-D-S42294A, ICD-10-D-S42295A, ICD-10-D-S42296A, ICD-10-D-S42301A, ICD-10-D-S42302A, ICD-10-D-S42309A, ICD-10-D-S42321A, ICD-10-D-S42322A, ICD-10-D-S42323A, ICD-10-D-S42324A, ICD-10-D-S42325A, ICD-10-D-S42326A, ICD-10-D-S42331A, ICD-10-D-S42332A, ICD-10-D-S42333A, ICD-10-D-S42334A, ICD-10-D-S42335A, ICD-10-D-S42336A, ICD-10-D-S42341A, ICD-10-D-S42342A, ICD-10-D-S42343A, ICD-10-D-S42344A, ICD-10-D-S42345A, ICD-10-D-S42346A, ICD-10-D-S42351A, ICD-10-D-S42352A, ICD-10-D-S42353A, ICD-10-D-S42354A, ICD-10-D-S42355A, ICD-10-D-S42356A, ICD-10-D-S42361A, ICD-10-D-S42362A, ICD-10-D-S42363A, ICD-10-D-S42364A, ICD-10-D-S42365A, ICD-10-D-S42366A, ICD-10-D-S42391A, ICD-10-D-S42392A, ICD-10-D-S42399A |
| Revision Arthroplasty | ICD-9-P-8197  ICD-10-P-0RWJ0J6, ICD-10-P-0RWJ0J7, ICD-10-P-0RWJ0JZ, ICD-10-P-0RWJXJ6, ICD-10-P-0RWJXJ7, ICD-10-P-0RWJXJZ, ICD-10-P-0RWK0J6, ICD-10-P-0RWK0J7, ICD-10-P-0RWK0JZ, ICD-10-P-0RWKXJ6, ICD-10-P-0RWKXJ7, ICD-10-P-0RWKXJZ  CPT-23473, CPT-23474 |
| Codes reported with decimal points removed, per PearlDiver coding format. | |
