# Supplementary material for: Total shoulder arthroplasty in patients with dementia or mild cognitive impairment
Source: JSES Int. 2023 Oct 7;8(1):159–66. doi: 10.1016/j.jseint.2023.09.004 (PMC10837705; doi:10.1016/j.jseint.2023.09.004)
Supplement: Supplementary Table S3 [file mmc3.docx]

| **Supplementary Table III: ICD-9, ICD-10 and CPT Codes Queried for Medical Complications** | |
| --- | --- |
| **Description** | **Codes Queried** |
| Acute MI | ICD-9-D-41001, ICD-9-D-41011, ICD-9-D-41021, ICD-9-D-41031, ICD-9-D-41041, ICD-9-D-41051, ICD-9-D-41061, ICD-9-D-41071, ICD-9-D-41081, ICD-9-D-41091  ICD-10-D-I2101, ICD-10-D-I2102, ICD-10-D-I2109, ICD-10-D-I2111, ICD-10-D-I2119, ICD-10-D-I2121, ICD-10-D-I2129, ICD-10-D-I213, ICD-10-D-I214, ICD-10-D-I219 |
| Acute Renal Failure | ICD-9-D-5845, ICD-9-D-5846, ICD-9-D-5847, ICD-9-D-5848, ICD-9-D-5849  ICD-10-D-N170, ICD-10-D-N171, ICD-10-D-N172, ICD-10-D-N178, ICD-10-D-N179 |
| Pulmonary Embolism | ICD-9-D-41511, ICD-9-D-41513, ICD-9-D-41519  ICD-10-D-I2692, ICD-10-D-I2699 |
| Sepsis | ICD-9-D-99591, ICD-9-D-99592  ICD-10-D-A400, ICD-10-D-A401, ICD-10-D-A403, ICD-10-D-A408, ICD-10-D-A409, ICD-10-D-A4101, ICD-10-D-A4102, ICD-10-D-A411, ICD-10-D-A412, ICD-10-D-A413, ICD-10-D-A414, ICD-10-D-A4150, ICD-10-D-A4151, ICD-10-D-A4152, ICD-10-D-A4153, ICD-10-D-A4159, ICD-10-D-A4181, ICD-10-D-A4189, ICD-10-D-A419, ICD-10-D-R6520 |
| Delirium | ICD-9-D-29011, ICD-9-D-2903, ICD-9-D-29041, ICD-9-D-29281, ICD-9-D-2930, ICD-9-D-2931, ICD-9-D-2939, ICD-9-D-78002, ICD-9-D-78009, ICD-9-D-78097  ICD-10-D-F05, ICD-10-D-R404, ICD-10-D-R4182 |
| Cerebrovascular Accident | ICD-9-D-43301, ICD-9-D-43311, ICD-9-D-43321, ICD-9-D-43331, ICD-9-D-43381, ICD-9-D-43391, ICD-9-D-43401, ICD-9-D-43411, ICD-9-D-43491  ICD-10-D-I6300, ICD-10-D-I63011, ICD-10-D-I63012, ICD-10-D-I63013, ICD-10-D-I63019, ICD-10-D-I6302, ICD-10-D-I63031, ICD-10-D-I63032, ICD-10-D-I63033, ICD-10-D-I63039, ICD-10-D-I6309, ICD-10-D-I6310, ICD-10-D-I63111, ICD-10-D-I63112, ICD-10-D-I63113, ICD-10-D-I63119, ICD-10-D-I6312, ICD-10-D-I63131, ICD-10-D-I63132, ICD-10-D-I63133, ICD-10-D-I63139, ICD-10-D-I6319, ICD-10-D-I6320, ICD-10-D-I63211, ICD-10-D-I63212, ICD-10-D-I63213, ICD-10-D-I63219, ICD-10-D-I6322, ICD-10-D-I63231, ICD-10-D-I63232, ICD-10-D-I63233, ICD-10-D-I63239, ICD-10-D-I6329, ICD-10-D-I6330, ICD-10-D-I63311, ICD-10-D-I63312, ICD-10-D-I63313, ICD-10-D-I63319, ICD-10-D-I63321, ICD-10-D-I63322, ICD-10-D-I63323, ICD-10-D-I63329, ICD-10-D-I63331, ICD-10-D-I63332, ICD-10-D-I63333, ICD-10-D-I63339, ICD-10-D-I63341, ICD-10-D-I63342, ICD-10-D-I63343, ICD-10-D-I63349, ICD-10-D-I6339, ICD-10-D-I6340, ICD-10-D-I63411, ICD-10-D-I63412, ICD-10-D-I63413, ICD-10-D-I63419, ICD-10-D-I63421, ICD-10-D-I63422, ICD-10-D-I63423, ICD-10-D-I63429, ICD-10-D-I63431, ICD-10-D-I63432, ICD-10-D-I63433, ICD-10-D-I63439, ICD-10-D-I63441, ICD-10-D-I63442, ICD-10-D-I63443, ICD-10-D-I63449, ICD-10-D-I6349, ICD-10-D-I6350, ICD-10-D-I63511, ICD-10-D-I63512, ICD-10-D-I63513, ICD-10-D-I63519, ICD-10-D-I63521, ICD-10-D-I63522, ICD-10-D-I63523, ICD-10-D-I63529, ICD-10-D-I63531, ICD-10-D-I63532, ICD-10-D-I63533, ICD-10-D-I63539, ICD-10-D-I63541, ICD-10-D-I63542, ICD-10-D-I63543, ICD-10-D-I63549, ICD-10-D-I6359, ICD-10-D-I636, ICD-10-D-I6381, ICD-10-D-I6389, ICD-10-D-I639, ICD-10-D-I6781, ICD-10-D-I97811, ICD-10-D-I97821 |
| Deep Vein Thrombosis | ICD-9-D-45340, ICD-9-D-45341, ICD-9-D-45342  ICD-10-D-I82401, ICD-10-D-I82402, ICD-10-D-I82403, ICD-10-D-I82409, ICD-10-D-I82411, ICD-10-D-I82412, ICD-10-D-I82413, ICD-10-D-I82419, ICD-10-D-I82421, ICD-10-D-I82422, ICD-10-D-I82423, ICD-10-D-I82429, ICD-10-D-I82431, ICD-10-D-I82432, ICD-10-D-I82433, ICD-10-D-I82439, ICD-10-D-I82441, ICD-10-D-I82442, ICD-10-D-I82443, ICD-10-D-I82449, ICD-10-D-I82451, ICD-10-D-I82452, ICD-10-D-I82453, ICD-10-D-I82459, ICD-10-D-I82461, ICD-10-D-I82462, ICD-10-D-I82463, ICD-10-D-I82469, ICD-10-D-I82491, ICD-10-D-I82492, ICD-10-D-I82493, ICD-10-D-I82499, ICD-10-D-I824Y1, ICD-10-D-I824Y2, ICD-10-D-I824Y3, ICD-10-D-I824Y9, ICD-10-D-I824Z1, ICD-10-D-I824Z2, ICD-10-D-I824Z3, ICD-10-D-I824Z9 |
| Pneumonia | ICD-9-D-4800, ICD-9-D-4801, ICD-9-D-4802, ICD-9-D-4803, ICD-9-D-4808, ICD-9-D-4809, ICD-9-D-481, ICD-9-D-4820, ICD-9-D-4821, ICD-9-D-4822, ICD-9-D-4823, ICD-9-D-48231, ICD-9-D-48232, ICD-9-D-48239, ICD-9-D-4824, ICD-9-D-48241, ICD-9-D-48242, ICD-9-D-48249, ICD-9-D-48281, ICD-9-D-48282, ICD-9-D-48283, ICD-9-D-48284, ICD-9-D-48289, ICD-9-D-4829, ICD-9-D-4830, ICD-9-D-4831, ICD-9-D-4838, ICD-9-D-4841, ICD-9-D-4843, ICD-9-D-4845, ICD-9-D-4846, ICD-9-D-4847, ICD-9-D-4848, ICD-9-D-485, ICD-9-D-486, ICD-9-D-4870  ICD-10-D-A3791, ICD-10-D-J09X1, ICD-10-D-J1001, ICD-10-D-J1108, ICD-10-D-J120, ICD-10-D-J121, ICD-10-D-J122, ICD-10-D-J1281, ICD-10-D-J1289, ICD-10-D-J129, ICD-10-D-J130, ICD-10-D-J150, ICD-10-D-J151, ICD-10-D-J153, ICD-10-D-J154, ICD-10-D-J156, ICD-10-D-J157, ICD-10-D-J159, ICD-10-D-J160, ICD-10-D-J168, ICD-10-D-J180, ICD-10-D-J181, ICD-10-D-J188, ICD-10-D-J189, ICD-10-D-J851 |
| Aspiration Pneumonia | ICD-9-D-5070, ICD-9-D-5071, ICD-9-D-5078, ICD-9-D-99732  ICD-10-D-J690, ICD-10-D-J691, ICD-10-D-J698 |
| UTI | ICD-9-D-5990  ICD-10-D-N390 |
| SNF Utilization | CPT-99301, CPT-99302, CPT-99303, CPT-99304, CPT-99305, CPT-99306 |
| ED Visitation | CPT-99281, CPT-99282, CPT-99283, CPT-99284, CPT-99285, CPT-G0380, CPT-G0381, CPT-G0382, CPT-G0383, CPT-G0384 |
| Mortality | ICD-9-D-7981, ICD-9-D-7982, ICD-9-D-7989  ICD-10-D-R99  CPT-T2042, CPT-T2043, CPT-T2044, CPT-T2045, CPT-T2046, CPT-G9751, CPT-G9812, CPT-G9852, CPT-G9855, CPT-99377, CPT-99378, CPT-G9473, CPT-G9475, CPT-G9476, CPT-G9477, CPT-G9478, CPT-G9479, CPT-G9524, CPT-G9525, CPT-G9687, CPT-G9688, CPT-G9690, CPT-G9691, CPT-G9692, CPT-G9693, CPT-G9694, CPT-G9700, CPT-G9702, CPT-G9707, CPT-G9709, CPT-G9710, CPT-G9714, CPT-G9715, CPT-G9718, CPT-G9720, CPT-G9723, CPT-G9725, CPT-G9740, CPT-G9741, CPT-G9761, CPT-G9768, CPT-G9857, CPT-G9809, CPT-G9858, CPT-M1022, CPT-Q5004, CPT-Q5005, CPT-Q5006, CPT-Q5007, CPT-Q5008, CPT-Q5010, CPT-S0255, CPT-S0271, CPT-S9126 |
| Codes reported with decimal points removed, per PearlDiver coding format. MI, Myocardial Infarction; UTI, Urinary Tract Infection; SNF, Skilled Nursing Facility; ED, Emergency Department. | |
